# Supplementary material for: On-demand transposition across light-matter interaction regimes in bosonic cQED
Source: Nat Commun. 2024 Jul 10;15:5816. doi: 10.1038/s41467-024-50201-7 (PMC11236968; doi:10.1038/s41467-024-50201-7)
Supplement: Supplementary file 1 — Supplementary Information [file 41467_2024_50201_MOESM1_ESM.pdf]

Supplementary Information: On-demand transposition across light-matter interaction regimes in bosonic cQED

Fernando Valadares,[1,*] Ni-Ni Huang,[1] Kyle Timothy Ng Chu,[1,2] Aleksandr Dorogov,[1]
Weipin Chua,[3] Lingda Kong,[1] Pengtao Song,[1] and Yvonne Y. Gao[1,3,†]

[1] *Centre for Quantum Technologies, National University of Singapore, Singapore*
[2] *Horizon Quantum Computing, Singapore*
[3] *Department of Physics, National University of Singapore, Singapore*

(Dated: June 27, 2024)

SUPPLEMENTARY NOTE 1 . DEVICE DESIGN & SYSTEM PARAMETERS

The device used in this work is based on standard bosonic cQED systems [1, 2], consisting of one high-Q cavity, one SQUID transmon, and a low-Q resonator for readout. We simulate the electromagnetic fields of the device using Ansys finite-element High-Frequency Simulation Software (HFSS), and obtain the Hamiltonian parameters using the energy participation ratio (EPR) approach [3]. The key system properties, such as the frequency of each circuit and the pair-wise nonlinear couplings between them, are iteratively refined to meet the target parameters. In this section, we describe the details on the design considerations and resulting properties of the main elements in the device, namely, the cavity, the SQUID transmon, the magnetic hose, and the coil.

A. Hamiltonian parameter optimisation

The design of Hamiltonian parameters takes into consideration the variable SQUID transmon frequency. Specifically, the transmon's design incorporates two critical frequency points: one optimized for strong interaction with the cavity and a second point where the systems weakly coupled. In the latter point, the transmon maintains substantial coupling with the readout resonator, allowing the measurement of its state with minimal disturbance to the cavity. Later, we incorporate other points for experiments reported in the main text demonstrating resonance and suppression of nonlinearities. We summarised the Hamiltonian parameters at selected flux points in Supplementary Table I, and represented the accessible coupling regimes in Supplementary Fig. 1.

**Points**	$\Delta/2\pi$ (MHz)	$\chi_{qc}^{\text{exp}}/2\pi$ (MHz)	$\omega_t^{\text{exp}}/2\pi$ (GHz)	$\omega_t^{\text{sim}}/2\pi$ (GHz)	$K^{\text{sim}}/2\pi$ (kHz)
**A**	35	1.67	5.705	5.696	44
**B**	63	0.94	5.677	5.662	6
**C**	101	0.57	5.639	5.635	1.9
**D**	146	0.29	5.594	5.547	0.19
**E**	196	0.18	5.544	5.492	0.09
**F**	596	0.05	5.144	5.214	0.005

Supplementary Table I. **Summary of key system parameters at selected flux points.** The simulated and measured main parameters, organized in ascending order of detuning Δ from the cavity frequency, at different flux points relevant to the experiments reported in the main text. For values of the cavity self-Kerr, numbers are quoted based on simulation results only as some of the smaller self-Kerr values cannot be precisely measured due to experimental limitations.

The transmon's maximum frequency is designed to be 6.5 GHz, strategically positioned between the frequencies of the cavity (5.74 GHz) and the readout resonator (6.9 GHz). This specific configuration enables the transmon to achieve resonance with the cavity within the tunable range to perform the vacuum Rabi experiment. In addition, the transmon is also consistently operated below the cavity frequency to avoid entering the straddling regime [4], which ensures optimal performance and suppresses undesired interactions.

The cavity, transmon, and readout resonator are capacitively coupled, with the coupling strength determined by the overlap between their electromagnetic fields. In practice, the coupling factor g between each of the elements mainly

* Corresponding author: fernando.valadares@u.nus.edu
† Corresponding author: yvonne.gao@nus.edu.sg

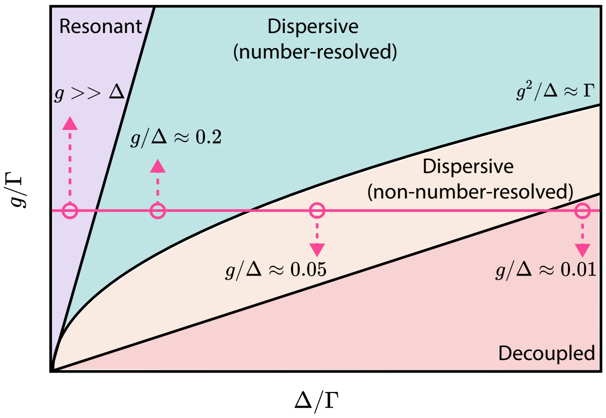

Supplementary Figure 1. **Phase diagram of light-matter coupling.** This phase diagram qualitatively represents the light-matter interaction regimes as a function of their frequency detuning Δ and coupling strength g normalized to the dominant decay rate Γ. The pink line shows the parameters that can be accessed with our system, highlighting points used for different experiments in this work. Purple region: The systems are resonant and undergo vacuum Rabi oscillations. Green (Orange) region: dispersive region, where the matter system frequency can (cannot) be resolved into separate spectral lines for each number of excitations of the light field. Light red region: the systems are effectively decoupled.

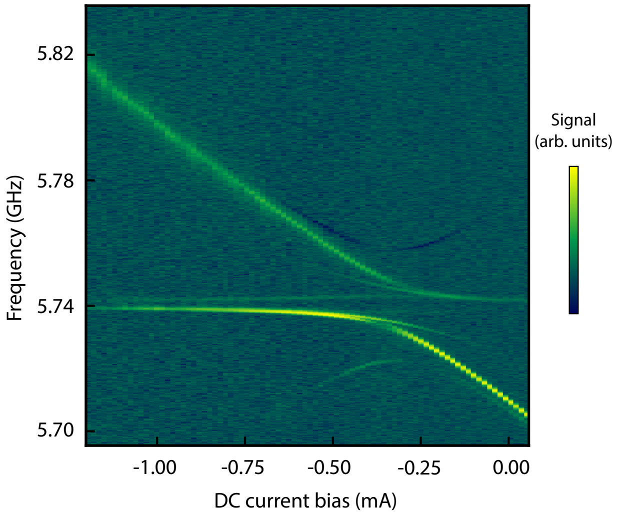

Supplementary Figure 2. **Vacuum Rabi avoided crossing.** As the transmon is tuned into to resonance with the cavity, anti-crossing with a gap $2g \approx 14.60\,\text{MHz}$ is observed. This is consistent with the simulated target parameter of $g = 7.44\,\text{MHz}$.

depends on geometrical parameters such as the sizes of the transmon capacitor pads and their distance from the cavity and the readout resonator. In our design, we chose a coupling factor of $g = 7.44\,\text{MHz}$ between the cavity and the transmon. This is sufficiently large for fast exchange of excitations between the two elements on the nanosecond timescale while allowing a safe distance between the magnetic hose and the cavity.

Experimentally, we observed consistent agreement between measured system parameters and design targets. For example, the vacuum Rabi splitting illustrated in Supplementary Fig. 2 indicates $g = 7.30\,\text{MHz}$. This reduced slightly to $g = 6.65\,\text{MHz}$ in subsequent thermal cycles, as measured from the rate of the vacuum Rabi oscillations shown in the main text (Fig. 2a). Similar agreement between experiment and simulation is observed across the full transmon frequency range.

B. Quality factor of the cavity

Apart from the Hamiltonian parameters, the coherence property of the cavity mode is also carefully considered in our design process. Our primary focus is minimizing cavity losses directly through the mu-metal layers of the magnetic hose. For this particular analysis, we deliberately exclude both the transmon and the readout resonator from the simulation to eliminate potential indirect losses associated with cavity hybridization with other circuits.

The simulations show that the critical factor to be optimized is the distance between the cavity and the hose. Notably, the Q-factor experiences a decrease of roughly an order of magnitude for each millimetre of approximation. Thus, making this spacing long enough is critical for our device. However, the hose has to be aligned with the SQUID loop at the middle of the transmon. Thus, distancing the hose from the cavity means the transmon must also be placed further away.

Moreover, the transmon pads cannot be arbitrarily stretched to reach the cavity, as this would cause the transmon field to spread out of the chip and to couple to the loss channel of the hose. To enhance the cavity-transmon coupling, we employ a superconducting strip, approximately 2.85 mm long, strategically placed on both sides of the transmon, as shown in Supplementary Fig. 4a. We ensure that this strip is short enough that it does not introduce any resonant mode that is close in frequency to the other elements. The strips effectively guide the transmon field to the cavity and readout resonator modes, recovering their coupling without requiring them to be in close proximity.

In the finalised design, the center of the hose is placed approximately 3.65 mm away from the edge of the cavity, resulting in a simulated upper bound for the cavity lifetime of 1.6 ms. Experimentally, we conducted standard cavity T_1 measurements across multiple thermal cycles, observing T_1 to range from $100\,\mu$s to $250\,\mu$s. This is mainly limited by the poor coherence times of the transmon ($T_1 \approx 15\,\mu$s, $T_2 \approx 0.5$-$4\,\mu$s). In addition, we also observed that the measured cavity T_1 does not depend on the flux applied to the coil, indicating that the introduction of the magnetic flux to the system is not the dominant loss in our device.

C. Magnetic Hose

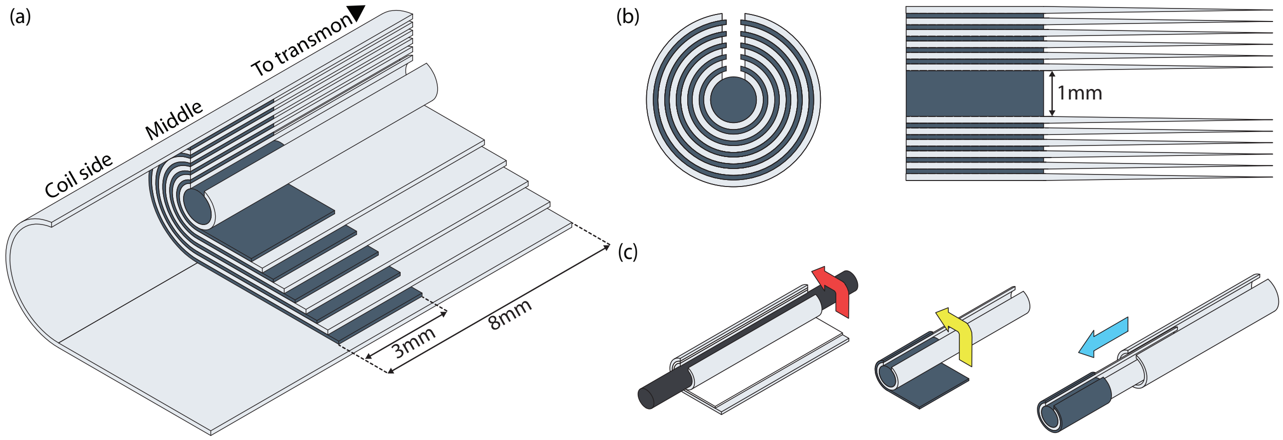

Supplementary Figure 3. **Assembly and design features of the hose** (a) Half-open view of assembled hose 3D model. Aluminium (mu-metal) layers are shown in light (dark) gray. The 3D model depicts the hose in three distinct sections: "Coil side", where the coil is inserted; the "Middle" section, where the mu-metal is placed to ensure high magnetic field transferability and hold the inner aluminium layers; and the output, labeled as "To transmon", made exclusively of aluminium layers and functioning as a high-pass filter. (b) Cross-section and side cut of the hose showing adaptations to prevent superconducting loops. In the left picture, the mu-metal layers are shown to have a smaller cut than the aluminium layers, providing protection against aluminium-aluminium contact. In the right picture, the aluminium layers are shown to be made thin at the output end to make the spacing between layers larger. (c) Hose assembly. First step (red arrow): aluminium layers are bent into cylinders. The first layer is bent around a placeholder 1 mm-diameter rod. Subsequent layers use paper (colored as white) as placeholders for mu-metal, matching the 0.1 mm thickness. After all aluminium layers are bent, the placeholders are removed and the layers disassembled. Second step (yellow arrow): the first aluminium layer is assembled around the mu-metal core. The first mu-metal layer is bent around the structure. Third step (blue arrow): the next aluminium layer is assembled to the structure. The second and third steps are repeated with every layer until the hose is assembled.

Conceptually, we can divide the hose into three sections. The input section, defined by the outer aluminium layer,

allows for the insertion of the small coil which generates the magnetic field traveling through the hose. This section can be elongated until it protrudes out of the device, creating a surface to be clamped from the outside, as depicted in Supplementary Fig. 3a and labeled “Coil side”. Next is the middle section, which houses aluminium, mu-metal layers, and the core wire, focusing on high transferability of the magnetic field due to the high magnetic permeability of the mu-metal. However, the properties of the mu-metal are very fragile, and its quality significantly deteriorates during the hose manufacturing. Consequently, this section is intentionally kept as short as possible, as illustrated in Supplementary Fig. 3a and labeled “Middle”.

The output section, depicted in Supplementary Fig. 3a and labeled “To transmon”, consists solely of aluminium layers, with a gap of 0.1 mm between each of them. Functioning as a high-pass filter, this section aims to attenuate any electromagnetic field emanating from the output end of the hose. As the hose guides flux to the transmon, the filter serves to prevent the circuit’s field from interacting with the lossy mu-metal layers. The length of this section is critical: if it is excessively long, the magnetic field provided to the transmon may be insufficient. Conversely, if it is too short, the filter may fail to effectively isolate the mu-metal from the transmon and cavity, thereby affecting their coherence times.

If there are any superconducting loops in the hose, the input magnetic field will generate stable supercurrents that oppose the field. Preventing superconducting loops requires careful adjustments to the hose so the aluminium layers don’t come in contact with each other. Firstly, in the middle section, widening the mu-metal layers slightly beyond the aluminium layers helps prevent the outer layers from folding into the inner layers, as illustrated in Supplementary Fig. 3b. In the output section, using sandpaper to reduce the thickness of the aluminium layers, from 0.15 mm to 0.06 mm - 0.08 mm, increases the space between layers, lowering the risk of contact. During the process of folding the aluminium layers into cylinders, it is critical to ensure that they are made as straight as possible to prevent any bending into each other at the output. These considerations collectively contribute to minimizing the possibility of unwanted superconducting loops.

The actual magnetic hose used in this device is designed and implemented based on the techniques outlined in Ref. [5] with the additional considerations outlined above. The procedure for making a hose starts with cutting five mu-metal rectangular pieces from a 0.1 mm-thick sheet and six aluminium pieces from a 0.15 mm-thick sheet, colored as dark and light gray, respectively, as shown in Supplementary Fig. 3a. Then, a mu-metal core is cut from a wire with a diameter of 1 mm. These sheets are systematically layered around the core, with alternating aluminium and mu-metal layers. Precise calculations of the width of each layer ensure that each layer covers the perimeter of the underlying layer, leaving a 0.5 mm gap to prevent superconducting loops.

All mu-metal pieces are cut to 3 mm in length, striking the balance of being the shortest length to suppress losses while still being easily manageable for handling. The five innermost aluminium layers are 8 mm in length. They fully cover the mu-metal layers, with a 3 mm section overlap between them and the remaining 5 mm extending into the vacuum. The outermost aluminium layer, elongated to 25 mm, accommodates the insertion of a coil, as illustrated in the Fig. 2a(i) in the main text.

D. Design and implementation of the coil

To ensure fast response to the input current, we need a relatively small coil. We crafted a disk-shaped coil (illustrated in the Fig. 2a(i) in the main text and Supplementary Fig 4b) by winding 19 to 21 turns of a 0.05 mm-diameter superconducting wire up to a maximum diameter of 3.4 mm. Due to the small dimensions, the coil must be crafted under a microscope with the aid of tweezers. To enhance field strength, we used a double coil configuration. This comes at the cost of decreasing the coil bandwidth, but its response is still fast enough to enact nonadiabatic dynamics in the vacuum Rabi experiment. After winding, the two layers are stacked with the aid of epoxy and then affixed to a dielectric support. The support is also affixed to the outer layer of the hose to improve thermalisation. The coil lead wires are soldered to an SMA adaptor that connects to the RF flux line.

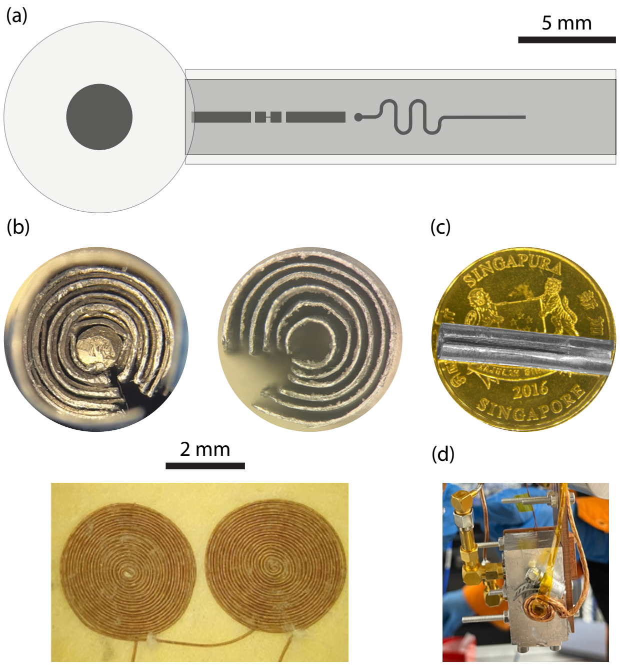

Supplementary Figure 4. **Full device illustration**. (a) The system illustrated from a top view, showing the coaxial stub cavity (left), transmon with the extended superconducting strips (middle), and meandering readout resonator (right). (b) Pictures of the magnetic hose and coil to scale. The left image shows the input side of the hose, where both aluminium and mu-metal layers can be seen. The right image displays the output end, where only aluminium layers are present. To the best of our knowledge, the aluminium layers don't come in contact with each other, and the 0.5 mm cut remains open along the hose. The bottom picture shows the double coil used as field source. After being wound, the two layers are glued together with epoxy and assembled to the hose. (c) Example of a hose compared with a Singapore coin of a diameter of 21 mm. (d) Picture of the device showing the thermalisation of the hose using copper braids.

SUPPLEMENTARY NOTE 2 . WIRING & MEASUREMENT SETUP

A. Cryogenic configuration

Our device is machined out of pure aluminium with a tunable transmon realised as a DC-SQUID featuring two double-angle evaporated Josephson junctions (each with inductance of 13 nH). The on-chip features are fabricated on a sapphire substrate, which is then inserted into the high-purity (5N) aluminium package. The whole device is housed in a standard Cryoperm shield to protect the quantum circuit from external electromagnetic noise.

The system is thermally anchored to the base stage of the dilution refrigerator. To improve the thermalisation of the flux line, OFHC copper braids (as depicted in Supplementary Fig. 4d) are used to help dissipate heat from the current applied to the coil. A short section of a copper braid connects the magnetic hose directly to the copper brackets that are affixed to the base plate of the fridge. Additionally, the two solder points along the line are directly thermally anchored to the base plate (one at the first eccosorb filter after the coil and another at the bias tee). With these procedures, we are able to access the full tunability range of the transmon without raising the mixing chamber temperature beyond 10 mK.

The readout of the transmon state is performed with a standard reflection setup. The output signal is amplified by a phase-sensitive SNAIL Parametric Amplifier (SPA) [6, 7] at the lower stage of the refrigerator. The signal is further amplified at the 4 K stage by a low-noise HEMT amplifier (LNF-LNC4_8C), and by a 26 dB amplifier at room temperature (ZVA-183-S+) before demodulation.

B. Room temperature configuration

The transmon and cavity are controlled by a fast field-programmable gate array (FPGA) system from Quantum Machines (QM). The Digital-to-Analogue Converter (DAC) port of the FPGA is used to create signals within a 250 MHz bandwidth. The control and probing pulses of the system are produced by upconverting intermediate frequency (IF) FPGA signals by IQ mixing with local oscillators (LO).

The DAC is also connected to an RF flux line that carries fast flux pulses to the coil. The coil is also biased with a stable current provided by a Yokogawa DC source, which is combined to the RF flux line using a bias tee (Marki Microwave BT0040) at the base plate of the fridge. For certain experiments, the fast flux signal is also amplified by a room-temperature amplifier (Stanford Research Systems SR445A) to provide a larger range of drive amplitudes to the coil. To mitigate high-frequency current noise at the coil, low-pass filters with 1.3 GHz cutoff (Minicircuits VLFX-1300) are placed on the flux line.

The readout tone, SPA pump, as well as an IF tone are all generated by a phase-locked multi-channel signal generator (AnaPico APMS-ULN) to ensure phase stability. The returning signal is downconverted through an IR mixer with an IF frequency of 250 MHz. This specific frequency is chosen to align with the clock cycle of the FPGA, further ensuring that no additional phase is introduced between each readout cycle. The downconverted signal is subsequently sampled in an Analogue-to-Digital Converter (ADC) block of the FPGA.

The schematic of the setup is summarised in Supplementary Fig. 5.

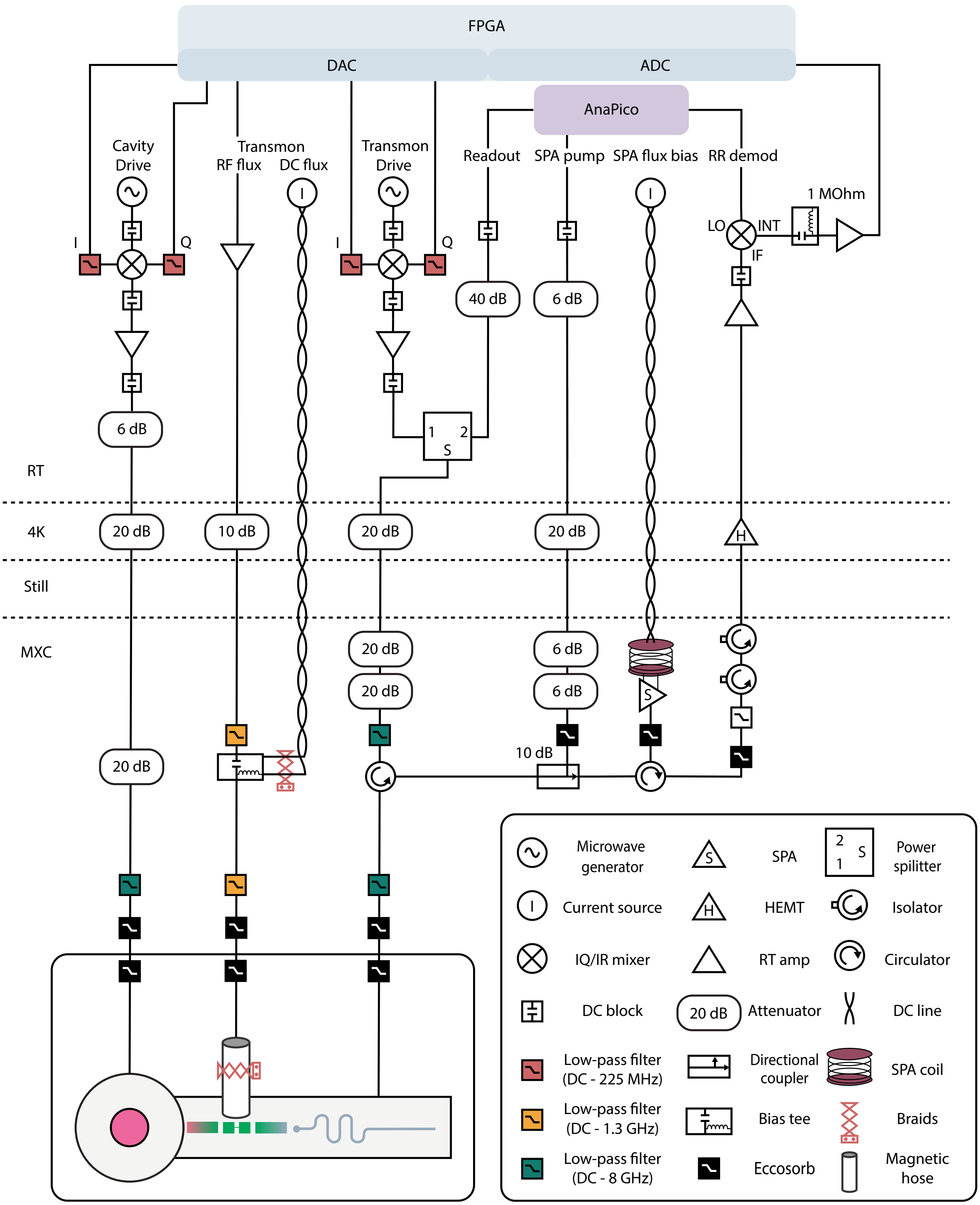

Supplementary Figure 5. **Experimental setup.** The RF control lines are configured with a standard set of filters, including a low-pass filter (Mini-Circuits BLP-250+ MHz) located outside the refrigerator and followed by a room-temperature amplifier Mini-Circuits ZVA-183-S+, working at GHz frequencies. Moreover, Eccosorb and standard low-pass filters (Hermerc System 8 GHz) are also incorporated within the mixing chamber. The DC supplies to the coil are provided through built-in DC lines in the fridge, and combined with the RF drives via a cryogenic bias tee.

SUPPLEMENTARY NOTE 3 . FLUX PULSE PRE-DISTORTION

The transmon frequency $\omega_t(t)$ is controlled by the current $I_c(t)$ at the coil as a function of time, according to the relation

$$\omega_t(t) \approx (\omega_t^{\max} + \alpha) \sqrt{\left| \cos\left(\frac{\pi k I_c}{\Phi_0}\right) \right|} - \alpha. \tag{1}$$

The coil is driven through the RF line connected to the room-temperature electronics (Supplementary Fig. 5). The FPGA sends programmable flux pulses $V_f(t)$ to the line, which can be assumed to be linearly related to the coil current through the relation

$$\tilde{I}_c(s) = H_{\text{line}}(s) \tilde{V}_f(s), \tag{2}$$

where $\tilde{I}_c$ and $\tilde{V}_f$ are the Laplace representations of the current and voltage waveforms and H_{line} is a function that accounts for the linear response of the RF flux line.

The term, H_{line}, includes the effective impedance of all components along the line, such as the bias tee, the filters, and the coil itself, leading to a complex distortion of the current waveform. These distortions have critical experimental consequences. Instability of the transmon frequency at the microsecond timescale causes imprecision in longer gates such as frequency-selective π-pulses. Also, the frequency transition speed can be slowed down by the presence of low-pass filtering, compromising experiments that require nonadiabatic frequency switching such as vacuum Rabi oscillations. The solution to accurately control of $\omega_t(t)$ is to program a predistorted flux pulse V_f that takes the line response H_{line} into account and generates the intended I_c.

To achieve the predistortion of the flux pulse, we first characterize the function H_{line} by measuring the current response to a step flux pulse $I_{\text{step}}(t)$. The step response encodes all information about H_{line} and is obtained with a pi-scope experiment (Supplementary Fig. 6a). This method resolves the transmon frequency trajectory along the step flux pulse by executing multiple transmon spectroscopies as a function of time. The length of the spectroscopy probe pulse sets the time resolution of the experiment (we use 16 ns constant pulses). The spectroscopy data is analyzed at each point of time to find the transmon frequency $\omega_t(t)$, which is used to obtain $I_{\text{step}}(t)$ using Supplementary Eq. 1.

From the step response, we train a set of digital infinite impulse response (IIR) and finite impulse response (FIR) filters that, combined, approximate the inverse function H_{line}^{-1} [8]. Any target current trajectory I_{target} can then be faithfully replicated by programming a predistorted flux pulse with the formula $\tilde{V}_f = H_{\text{line}}^{-1} \tilde{I}_{\text{target}}$.

By successfully employing this method, we reach accurate control of the transmon trajectory, necessary for controlling the cavity-transmon interactions reliably. Here, we describe in more detail the formulas and the procedures involved in training the IIR and FIR filters.

A. IIR filters

Supplementary Fig. 6b shows $I_{\text{step}}(t)$ obtained by measuring the $\omega_t(t)$ response to a step flux pulse with pi-scope and inverting Supplementary Eq. 1. The trajectory shows a multi-exponentially decaying current, along with a rising edge that also shows exponential behaviour. Our first distortion-correcting strategy is to build a set of IIR filters that are each tailored to invert a single exponential trend in the trajectory.

We can see how this works in a simplified example. Assume a step response of the form $I_{\text{step}} = \left(A + Be^{-t/\tau}\right) u(t)$, where $u(t)$ is the Heaviside function. The corresponding transfer function can be calculated by taking the Laplace transform of I_{step} and using Supplementary Eq. 2

$$H_{\text{exp}} = \left(\frac{A}{s} + \frac{B\tau}{1 + s\tau}\right) \tilde{V}_f^{-1}, \tag{3}$$

where $\tilde{V}_f = 1/s$ for the step response. The inverse of H_{exp} is simply

$$H_{\text{exp}}^{-1} = \frac{1 + s\tau}{A + s\tau\,(A + B)}. \tag{4}$$

In practice, we want to build discrete-time predistortion filters that can be programmed in the FPGA. To discretize H_{exp}^{-1}, we apply the bilinear transformation with a timestep T_s:

$$s \leftarrow \frac{2}{T_s} \frac{1 - z^{-1}}{1 + z^{-1}}. \tag{5}$$

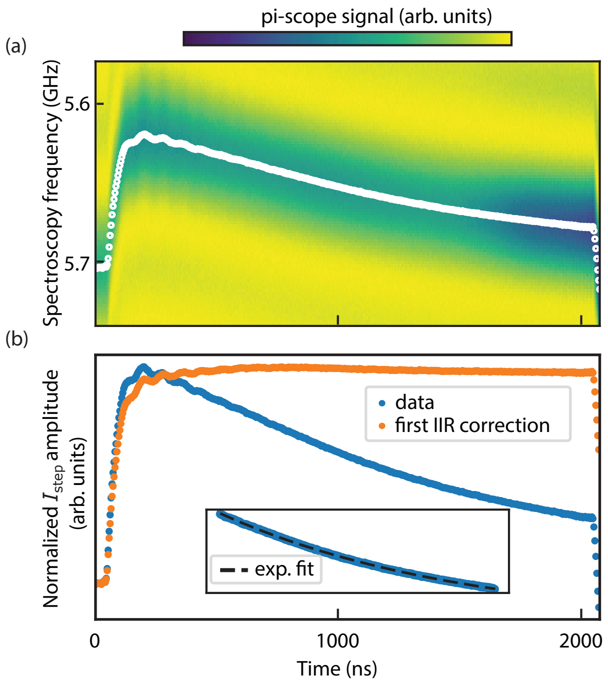

Supplementary Figure 6. **Flux pulse predistortion.** (a) Pi-scope experiment consisting of a time-dependent transmon spectroscopy during the application of a step flux pulse. $\omega_t(t)$ is extracted at each point of time (white dots). (b) Example of a first IIR correction. The current response is obtained from the transmon frequency data. An IIR filter is build from an exponential fit and used to correct the current response, making it more stable for longer periods of time.

This results in the digital predistortion IIR filter

$$H_{\text{IIR}}(z) = \frac{b_0 + b_1 z^{-1}}{1 - a_1 z^{-1}}, \tag{6}$$

where $a_1 = (2A\tau + 2B\tau - AT_s)/\lambda$, $b_0 = (2\tau + T_s)/\lambda$, $b_1 = (-2\tau + T_s)/\lambda$ and $\lambda = (2A\tau + 2B\tau + AT_s)$.

We correct I_{step} iteratively by fitting it to exponential functions to extract IIR filters according to Supplementary Eq. 6, as shown in Supplementary Fig. 6b. After the first filter $H_{\text{IIR},1}$ is extracted, the I_{step} data is corrected before training the next pulse. This can be done by repeating the pi-scope experiment using a step input predistorted by $H_{\text{IIR},1}$. Alternatively (as extracting a new pi-scope measurement is time-consuming), the expected correction to I_{step} can be calculated by numerically applying $H_{\text{IIR},1}$. This process is then repeated until there is no evident exponential trend in I_{step}.

Even after concluding the IIR corrections, I_c might still present oscillatory behavior close to the pulse edges (such ripples can already be seen in Supplementary Fig. 6). Next, we discuss how these can be corrected with an FIR filter.

B. FIR filter

FIR filters are used to correct the fast ripples that remain after the IIR filter corrections. In the time-domain, Supplementary Eq. 2 becomes the convolution

$$I_c = h * V_f + n, \tag{7}$$

where h is the impulse response of the system, and n is the noise present in the measurement of I_c. If V_f is a step function, h can be calculated using the step response I_{step} by

$$h = \frac{dI_{\text{step}}}{dt}. \tag{8}$$

As in the previous section, we want to find the inverse h_{inv}. However, due to the presence of noise, it is not straightforward to invert the convolution in Supplementary Eq. 7.

Instead, we explore the property that the convolution $h * h_{\text{inv}} = \delta$, where δ is the Dirac delta (or Kronecker delta in the case of discrete-time functions). If the system input is $V_f = h_{\text{inv}}$, then the output is δ up to measurement noise. So h_{inv} can be found numerically through the optimisation problem

$$h_{\text{inv}} = \underset{x}{\operatorname{argmin}}\{||h * x - \delta||\}, \tag{9}$$

where $||\cdot||$ represents the L_2 norm.

However, in this formulation, the solution h_{inv} will still be sensitive to the noise that I_{step} carries into h from Supplementary Eq. 8. To reduce the effect of noise, we use a cost function $\alpha||Dx||$:

$$h_{\text{inv}} = \underset{x}{\operatorname{argmin}}\{||h * x - \delta|| + \alpha||Dx||\}, \tag{10}$$

where D is a differentiation operator and α is the weight of the cost function. This optimisation penalises fast-oscillating values of x that result from over-fitting the noise.

Once the h_{inv} is known, it can be straightforwardly implemented with an FIR filter. In discrete-time formulation, an FIR filter has the general form

$$y[n] = b * x = \sum_{i=0}^{N} b[n]x[n-i], \tag{11}$$

where b_i are the coefficients of the filter. By simply assigning $b[n] = h_{\text{inv}}[n]$, the filter transforms a target pulse x into the predistorted pulse y. Together with the previously trained IIR filters, this concludes the flux predistortion.

SUPPLEMENTARY NOTE 4 . DATA ANALYSIS

A. Measurement and Post-Processing

1. *Wigner function tomography*

In the strong dispersive regime, we implement Wigner functions to the cavity state ρ by performing a set of cavity displacements $\hat{D}(\beta)$ and measuring the photon-number parity $\hat{\mathcal{P}}$, which leads to

$$W(\beta) = \frac{2}{\pi}\langle\hat{\mathcal{P}}\rangle = \frac{2}{\pi}\mathrm{Tr}(\hat{D}^{\dagger}(\beta)\rho\hat{D}(\beta)\hat{\mathcal{P}}). \tag{12}$$

The parity measurement involves interleaving a conditional cavity π-phase shift between two $R_y(\frac{\pi}{2})$ rotations, with $R_y(\frac{\pi}{2})$ representing a $\frac{\pi}{2}$ rotation around the y-axis as shown in Fig. 3b in the main text.

The cross-Kerr effect between the readout mode and the storage cavity introduces distortions to the readout signal when large displacements occur in the storage cavity. In order to mitigate this, and also to convert the readout signal to parity, we implemented the approach described in Ref. [9, 10]. The idea is to perform two parity measurements for a vacuum state in the cavity, with the rotation axis of the second $\frac{\pi}{2}$ pulse to be $R_y(\frac{\pi}{2})$ and $R_y(-\frac{\pi}{2})$, respectively. These two measurements are one-dimensional cuts along $\mathrm{Im}[\beta] = 0$. The difference between them corresponds to the parity value, which returns $\langle\hat{\mathcal{P}}\rangle = \pm 1$ for an ideal system with no state preparation and measurement errors.

In practice, the measurement in our system has finite precision. The single-shot readout is performed using a SPA operating with 16 dB of gain in a phase sensitive regime. We use a square readout pulse with a length of 400 ns, and the signal is acquired over a duration of 1000 ns. With additional FPGA delays, the total readout time extends to 1.5 μs, leading to a readout fidelity of $1 - [P(e|g) + P(g|e)]/2 \approx 87.9\%$, where $P(i|j)$ is the probability of measuring the state $|i\rangle$ when having prepared the state $|j\rangle$.

Furthermore, due to the presence of the cross-Kerr effect, a Gaussian curve is fitted to the data to obtain key calibration parameters, including amplitude and offset, based on the vacuum data. We then use the parameters extracted from this procedure to calibrate the Fock state Wigner tomography data shown in Fig. 3c of the main text.

2. *Characteristic function tomography*

In the weak dispersive regime, we employ characteristic functions, defined as $C(\nu) = \mathrm{Tr}(\hat{D}(\nu)\rho)$, to characterize the cavity state ρ. The characteristic function, as depicted in Fig. 3b bottom in the main text, is implemented using the same procedure as in Ref. [11], which consists of a transmon $\frac{\pi}{2}$ pulse followed by an echo conditional displacement (ECD) gate and another $\frac{\pi}{2}$ pulse. The ECD gate is composed of four cavity displacements and one transmon π pulse. By choosing the phase of the second transmon $\frac{\pi}{2}$ pulse to be either 0° or 90°, we can measure either the real part, $\mathrm{Re}[C(\nu)]$, or imaginary part, $\mathrm{Im}[C(\nu)]$, of the characteristic functions $C(\nu)$.

We use the vacuum state to calibrate the characteristic function measurement by sweeping the amplitude of the ECD gate. This is a one-dimensional measurement along $\mathrm{Im}[(\nu)] = 0$ of the $\mathrm{Re}[C(\nu)]$ of the vacuum. The objective is to obtain a unit displacement amplitude with a targeted Gaussian standard deviation $\sigma = 1$. Using a Gaussian fit, we extract the main calibration parameters, including σ, offset, and amplitude, from the vacuum state and use them to calibrate the subsequent characteristic function measurements. In addition, we calibrate the reference frame of the transmon phase so the $\mathrm{Im}[C(\nu)]$ is null.

When measuring the characteristic function of relatively large coherent states ($|\alpha| \geq 2$), we observe an elevated background in certain regions of the phase space. We attribute these phenomena to the large displacements required to measure the characteristic function. When these displacements are along the original coherent state amplitude, we create a significant photon population in the cavity which likely causes other spurious dynamics in the system. We performed several control experiments to verify that this effect does not depend on the specific transmon pulse-sequence or the flux-pulses. Furthermore, it does not affect the features in the centre of the phase-space, which is the region where the key information about the coherent states is contained. Thus, in the final data in Fig. 4 of the main text, we calibrate the background to remove these spurious artifacts.

B. Fidelity estimation

The fidelities of the generated states are estimated by directly calculating the overlap integral of the data to the corresponding ideal Wigner function and characteristic function over the phase space. For a state with a characteristic

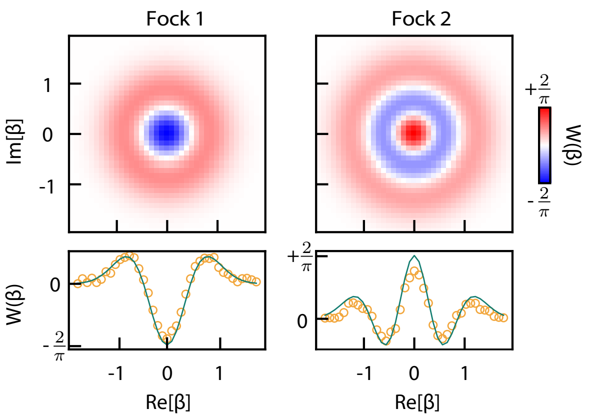

Supplementary Figure 7. **Comparison of simulated and measured Wigner functions of the Fock states.** Top: 2D plots of the simulated Fock $|1\rangle$ and $|2\rangle$, respectively. Bottom: line cuts along $\mathrm{Im}[\beta] = 0$ in the simulated results (green line) and the corresponding measured data (orange cirles). The plots show a consistent agreement between the experimental data and the simulated behavior.

function denoted as $C_{\mathrm{exp}}(\nu)$, the overlap to the ideal target state $C_{\mathrm{ideal}}(\nu)$ is calculated using the equation:

$$\mathcal{F}_{\mathrm{int}} = \frac{1}{\pi} \int C_{\mathrm{ideal}}(\nu) C^*_{\mathrm{exp}}(\nu) d^2\nu. \tag{13}$$

Similarly, for a state with a Wigner function $W_{\mathrm{exp}}(\beta)$, we compute the overlap to the ideal target state $W_{\mathrm{ideal}}(\beta)$ using

$$\mathcal{F}_{\mathrm{int}} = \frac{1}{\pi} \int W_{\mathrm{ideal}}(\beta) W_{\mathrm{exp}}(\beta) d^2\beta. \tag{14}$$

Since the acquired data is discrete, the integrals are converted to a summation. The calculated fidelities for the Wigner (characteristic) functions of the Fock states 1 and 2 are 91.1 (82.8) (%) and 66.6 (62.8) (%), respectively. The parity of each state is measured according to the centre of the Wigner function as $\mathcal{P}_1 \approx -0.90$ and $\mathcal{P}_2 \approx 0.76$.

The loss in fidelity is attributed to the imperfect preparation of $|e\rangle$ as the duration of the π-pulse used (80 ns) is significant compared to the transmon T_2 ($\approx$ 500 ns away from the sweet spot). State $|e\rangle$ is prepared twice during the creation of state $|2\rangle$, explaining further reduction in fidelity. This rate of decoherence is not a fundamental limitation of the present implementation, and it can be optimized by (1) reducing the large SQUID loop area, which is related to susceptibility of the circuit to magnetic noise, (2) improving the flux line filtering, and (3) adopting known noise-protection techniques such as using an asymmetric and/or concentric SQUID loop [12, 13].

We show the comparison between the 1D cuts in the ideal and measured Wigner function for the two states in Supplementary Fig. 7. While the features of the states are in close agreement, there is a reduction in the overall contrast in the data, consistent with the estimated fidelities.

C. Simulated cavity dynamics with different χ and K configurations

In this section, we compare the experimental data presented in Fig. 4 in the main text with the corresponding simulation results. To model the effects of cavity self-Kerr and the decoupling between the transmon and the cavity, we use a standard master equation simulation that closely follows the experimental protocols outlined in the main text. The simulation also accounts for the independently calibrated parameters for cavity and transmon decoherence at the specific flux points used in the experiment. The resulting simulated state, shown in Supplementary Fig. 8, is in close agreement with the data presented in the main text. This indicates that our experiment is faithfully reproducing the intended dynamics in the cavity.

D. Vacuum Rabi oscillations

We directly compare the observed vacuum Rabi oscillations between the cavity and the transmon with numerical simulations using the measured coupling strength $g = 6.65\,\mathrm{MHz}$. We observe close agreement between the simulation

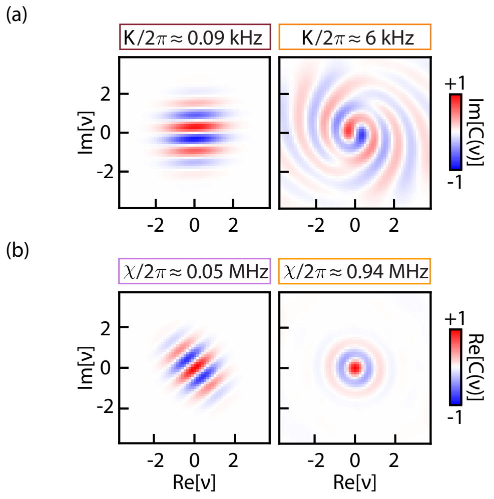

Supplementary Figure 8. **Simulation results of the suppression of undesired cavity dynamics.** (a) Mitigation of cavity self-Kerr. (b) Decoupling between transmon and cavity by turning off the dispersive interaction.

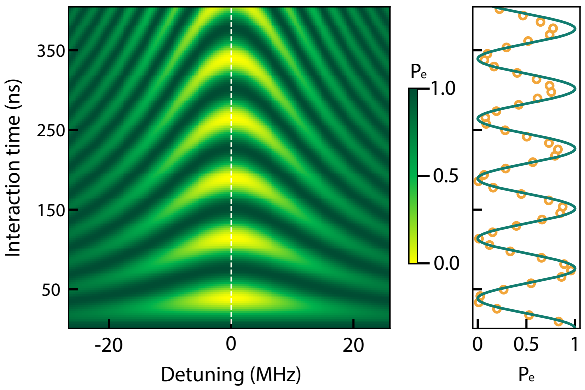

Supplementary Figure 9. **Simulation of vacuum Rabi oscillations between cavity and transmon.** Left: The coupling strength g corresponds to the value found in experiment. Vacuum Rabi oscillations are shown as a function of interaction time and detuning, with the white dashed line denoting zero detuning. The colorbar indicates the population of transmon excited state. Right: A cut along the y-axis of simulated behaviour at zero detuning (green line) are compared to the experimental data (orange circles).

(shown in Supplementary Fig. 9) and the measurement results shown in Fig. 3a in the main text.

The 1D cut along the y-axis shows the periodic oscillation that corresponds to the exchange of a single excitation between the transmon and the cavity. To account for the finite readout fidelity, we linearly transform the experimental points to map the highest observed transmon excited state population to 1 and the lowest to 0. The experimental results show an exponentially decaying oscillation due to the finite coherence times of the system. The amplitude of vacuum Rabi oscillation decays with a characteristic time of $\tau \approx 1.5\,\mu$s, which is mainly limited by the decoherence time of the transmon but is increased due to hybridization with the cavity.

[1] A. Blais, R.-S. Huang, A. Wallraff, S. M. Girvin, and R. J. Schoelkopf, Cavity quantum electrodynamics for superconducting electrical circuits: An architecture for quantum computation, Physical Review A **69**, 062320 (2004).
[2] S. M. Girvin, Circuit qed: superconducting qubits coupled to microwave photons, Quantum machines: measurement and control of engineered quantum systems , 113 (2014).
[3] Z. K. Minev, Z. Leghtas, S. O. Mundhada, L. Christakis, I. M. Pop, and M. H. Devoret, Energy-participation quantization of josephson circuits, npj Quantum Information **7**, 131 (2021).
[4] P. Krantz, M. Kjaergaard, F. Yan, T. P. Orlando, S. Gustavsson, and W. D. Oliver, A quantum engineer's guide to superconducting qubits, Applied Physics Reviews **6** (2019).
[5] O. Gargiulo, S. Oleschko, J. Prat-Camps, M. Zanner, and G. Kirchmair, Fast flux control of 3d transmon qubits using a magnetic hose, Applied Physics Letters **118** (2021).
[6] N. Frattini, V. Sivak, A. Lingenfelter, S. Shankar, and M. Devoret, Optimizing the nonlinearity and dissipation of a snail parametric amplifier for dynamic range, Physical Review Applied **10**, 054020 (2018).
[7] A. Dorogov, G. Fedorov, D. Kalacheva, A. Dmitriev, A. Bolgar, N. Abramov, and O. Astafiev, Application of a broadband josephson parametric amplifier, St. Petersburg State Polytechnical University Journal. Physics and Mathematics **15**, 352 (2022).
[8] J. Butscher, *Shaping of Fast Flux Pulses for Two-Qubit Gates*, Master's thesis, ETH Zurich (2018).
[9] L. Sun, A. Petrenko, Z. Leghtas, B. Vlastakis, G. Kirchmair, K. Sliwa, A. Narla, M. Hatridge, S. Shankar, J. Blumoff, *et al.*, Tracking photon jumps with repeated quantum non-demolition parity measurements, Nature **511**, 444 (2014).
[10] B. Vlastakis, G. Kirchmair, Z. Leghtas, S. E. Nigg, L. Frunzio, S. M. Girvin, M. Mirrahimi, M. H. Devoret, and R. J. Schoelkopf, Deterministically encoding quantum information using 100-photon schrödinger cat states, Science **342**, 607 (2013).
[11] P. Campagne-Ibarcq, A. Eickbusch, S. Touzard, E. Zalys-Geller, N. E. Frattini, V. V. Sivak, P. Reinhold, S. Puri, S. Shankar, R. J. Schoelkopf, *et al.*, Quantum error correction of a qubit encoded in grid states of an oscillator, Nature **584**, 368 (2020).
[12] M. Hutchings, J. Hertzberg, Y. Liu, N. Bronn, G. Keefe, M. Brink, J. M. Chow, and B. Plourde, Tunable Superconducting Qubits with Flux-Independent Coherence, Physical Review Applied **8**, 044003 (2017), publisher: American Physical Society.
[13] J. Braumüller, M. Sandberg, M. R. Vissers, A. Schneider, S. Schlör, L. Grünhaupt, H. Rotzinger, M. Marthaler, A. Lukashenko, A. Dieter, A. V. Ustinov, M. Weides, and D. P. Pappas, Concentric transmon qubit featuring fast tunability and an anisotropic magnetic dipole moment, Applied Physics Letters **108**, 032601 (2016).
